# Atopic Dermatitis or Subacute Cutaneous Lupus: Resolving a Diagnostic Challenge Through Clinicopathologic Correlation

**DOI:** 10.7759/cureus.117337

**Published:** 2026-10-01

**Authors:** Ana Sophia Ortiz Robles, Norma Edith Torres Gudiño, Flavio Antonio Sarabia Alba, Andreina Moreno Roman, Victoria Lopez Estudillo

**Affiliations:** 1 Internal Medicine, Hospital General Dr. Darío Fernández Fierro, Institute for Social Security and Services for State Workers, Mexico City, MEX; 2 Rheumatology, Hospital General Dr. Darío Fernández Fierro, Institute for Social Security and Services for State Workers, Mexico City, MEX; 3 Orthopaedics, Instituto Nacional de Rehabilitación Luis Guillermo Ibarra Ibarra, Mexico City, MEX

**Keywords:** atopic dermatitis, drug-induced liver injury, hemophagocytic syndrome, interface dermatitis, subacute cutaneous lupus erythematosus

## Abstract

This report presents a 44-year-old Mexican woman with hypothyroidism and atopic dermatitis who developed severe mixed-pattern hepatotoxicity after a 10-day course of an over-the-counter joint supplement (Ismerely FTX Plus) of incompletely disclosed composition. A public US Food and Drug Administration (FDA) Medication Health Fraud Notification confirmed the product was adulterated with undeclared dexamethasone phosphate and diclofenac. The clinical course was complicated by probable secondary hemophagocytic syndrome, with a peak ferritin of 19,114.9 ng/mL (93 times the upper reference limit), resolving spontaneously without immunosuppression after discontinuation of the causative agent. The rarity of this case lies in the convergence of an unconventional trigger, a hemophagocytic lymphohistiocytosis (HLH)-like response, and spontaneous resolution in a host with pre-existing autoimmune vulnerability.

The patient presented with five days of intermittent fever up to 39.4°C and severe right upper quadrant pain, followed by fatigue, hair changes, and cutaneous lesions of the right upper extremity and face. Outpatient studies showed negative viral hepatitis serologies and splenomegaly. Hospital evaluation demonstrated severe mixed-pattern transaminasemia (alanine aminotransferase (ALT) 899 IU/L, aspartate aminotransferase (AST) 1,063 U/L, R factor 4.8), mild bilineage cytopenia (leukopenia 2.96 × 10³/µL, driven by lymphopenia rather than neutropenia, and mild thrombocytopenia 118 × 10³/µL, with normal hemoglobin), elevated inflammatory markers, and hypertriglyceridemia. Autoimmune testing showed a true-positive antinuclear antibody (ANA) titer of 1:80 (homogeneous/nuclear) and borderline anti-smooth muscle antibodies (1:100), with all other lupus-specific antibodies negative. Examination revealed periarticular papulosquamous lesions with mild edema on the right arm and elbow and bilateral malar erythema sparing the nasolabial folds. Skin biopsy showed spongiotic dermatitis with interface vacuolar degeneration and a subcorneal blister pattern, without vasculitis or tissue eosinophilia.

Absent eosinophilia argued against drug reaction with eosinophilia and systemic symptoms (DRESS), while negative lupus-specific serologies and unmet classification criteria argued against subacute cutaneous lupus erythematosus (SCLE) or systemic lupus erythematosus (SLE). The most coherent interpretation was drug- or supplement-induced interface dermatitis with idiosyncratic hepatotoxicity, plausibly mediated by cytotoxic T-cell activity against keratinocytes and hepatocytes modified by undisclosed corticosteroid and nonsteroidal anti-inflammatory drug (NSAID) metabolites. Transaminases, ferritin, and cytopenias improved progressively: ALT and AST declined approximately 43% and 62% within five days, with ALT reaching a 70% decline by discharge (hospital day 9), and ferritin declining 93% to 1,309.7 ng/mL at last determination (outpatient follow-up, day 17), in parallel with resolution of cutaneous lesions after supplement discontinuation alone. This case underscores the importance of clinicopathologic correlation over isolated findings and highlights adulterated supplements as an underrecognized trigger of combined hepatic, hematologic, and cutaneous immune-mediated injury.

## Introduction

Regulatory analysis has revealed that ostensibly "natural" joint or pain-relief supplements are adulterated with undisclosed pharmaceutical agents, including corticosteroids and nonsteroidal anti-inflammatory drugs, which can independently trigger hepatotoxic, immunologic, and cutaneous reactions [1,2]. Severe drug-induced liver injury (DILI) associated with over-the-counter dietary and herbal supplements is increasingly acknowledged, and its clinical presentation can be difficult to distinguish from viral, autoimmune, or infectious causes of acute hepatitis, particularly when the exact composition of the ingested product is unknown or misrepresented on its label [1,2].

A further diagnostic challenge develops when severe systemic inflammation is accompanied by extreme hyperferritinemia, raising concern for secondary hemophagocytic lymphohistiocytosis (HLH), a hyperinflammatory syndrome driven by uncontrolled macrophage activation that can be triggered by infections, malignancy, autoimmune disease, or drug reactions [3,4]. In parallel, the concurrent appearance of cutaneous lesions in a patient with a personal history of atopic dermatitis and borderline autoimmune serologies complicates the differential diagnosis further, since interface dermatitis on skin biopsy is not exclusive to cutaneous lupus erythematosus and can also be seen in drug reactions, including DRESS and other drug-induced interface dermatitides [5-7]. Interface dermatitis refers to an inflammatory pattern centered at the dermoepidermal junction, in which basal keratinocytes undergo vacuolar (hydropic) degeneration, a form of cell injury in which fluid accumulation creates clear vacuoles around or within basal cells, and this pattern, while classically associated with cutaneous lupus erythematosus, is not specific to it.

This case involves a 44-year-old woman with hypothyroidism and atopic dermatitis who developed severe hepatotoxicity, probable secondary HLH, and periarticular and facial cutaneous lesions after ingesting an adulterated joint supplement. The case demonstrates that clinicopathologic correlation, rather than reliance on any single laboratory or histopathologic finding, was essential to reach the most probable diagnosis and avoid unnecessary immunosuppressive therapy.

## Case presentation

A 44-year-old Mexican woman presented with fever, right upper quadrant abdominal pain, and dermatologic lesions on the right upper extremity. She denied a family history of autoimmune, hepatic, or hematologic disease. Her personal medical history included hypothyroidism, diagnosed approximately two years earlier and treated with levothyroxine at an unspecified dose, and atopic dermatitis of undocumented onset, previously managed by a private dermatologist with emollient cream. She had no other relevant medical or surgical history and no documented drug allergies.

The patient ingested an over-the-counter joint supplement (Ismerely FTX Plus) for 10 consecutive days before symptom onset, without medical prescription or knowledge of its full composition. A public FDA Medication Health Fraud Notification later confirmed the presence of dexamethasone phosphate and diclofenac in this product, which the label did not disclose [8]. This pattern of covert pharmaceutical adulteration is increasingly recognized among joint and pain-relief supplements [5,6].

During the first day of symptoms, she had sudden-onset intermittent fever up to 39.4°C, predominantly nocturnal, partially responsive to antipyretics, accompanied by chills and profuse diaphoresis, lasting approximately five days. Simultaneously, she developed sudden-onset colicky pain in the right upper quadrant, severe in intensity (9/10 on the visual analog scale), continuous, with diffuse irradiation, worsened by food intake and partially relieved by an antalgic position, associated with nausea without vomiting and without changes in bowel habits.

Given the persistence of symptoms, she initially sought care from a private physician, who prescribed ciprofloxacin 500 mg every 8 hours, paracetamol 500 mg every 8 hours, and magaldrate/simethicone, without clinical improvement. A week later, outpatient studies showed a negative viral panel for hepatitis A, B, and C, and abdominal ultrasound revealed splenomegaly, prompting referral and subsequent admission to Hospital General Darío Fernández Fierro, Institute for Social Security and Services for State Workers (ISSSTE).

Clinical findings

On physical examination at admission, the patient was in fair general condition, alert, oriented, and with a pained facial expression. Vital signs showed a temperature of 36.5°C, with heart rate and blood pressure within normal limits. The abdomen was tender to palpation in the right upper quadrant, without signs of peritoneal irritation. Splenomegaly was identified on examination, previously confirmed by abdominal ultrasound.

Dermatologic Findings: Face

A dermatosis was identified over the bilateral cheeks, malar region, nasolabial folds, and nasal dorsum, consisting of diffuse pink-red erythema and erythematous macules with poorly defined borders, bilateral and symmetric in distribution, extending from the malar region toward the nasolabial folds with partial sparing of the nasolabial sulcus (Figure 1). The surface was smooth, without scaling, crusting, atrophy, or ulceration, and no secondary lesions were identified. The bilateral, symmetric distribution over the malar region and nasal dorsum, with relative sparing of the nasolabial fold, configured a morphologic pattern consistent with a butterfly-shaped (malar) rash, a characteristic, though not exclusive, finding of systemic lupus erythematosus (Table 1).

**Figure 1:**
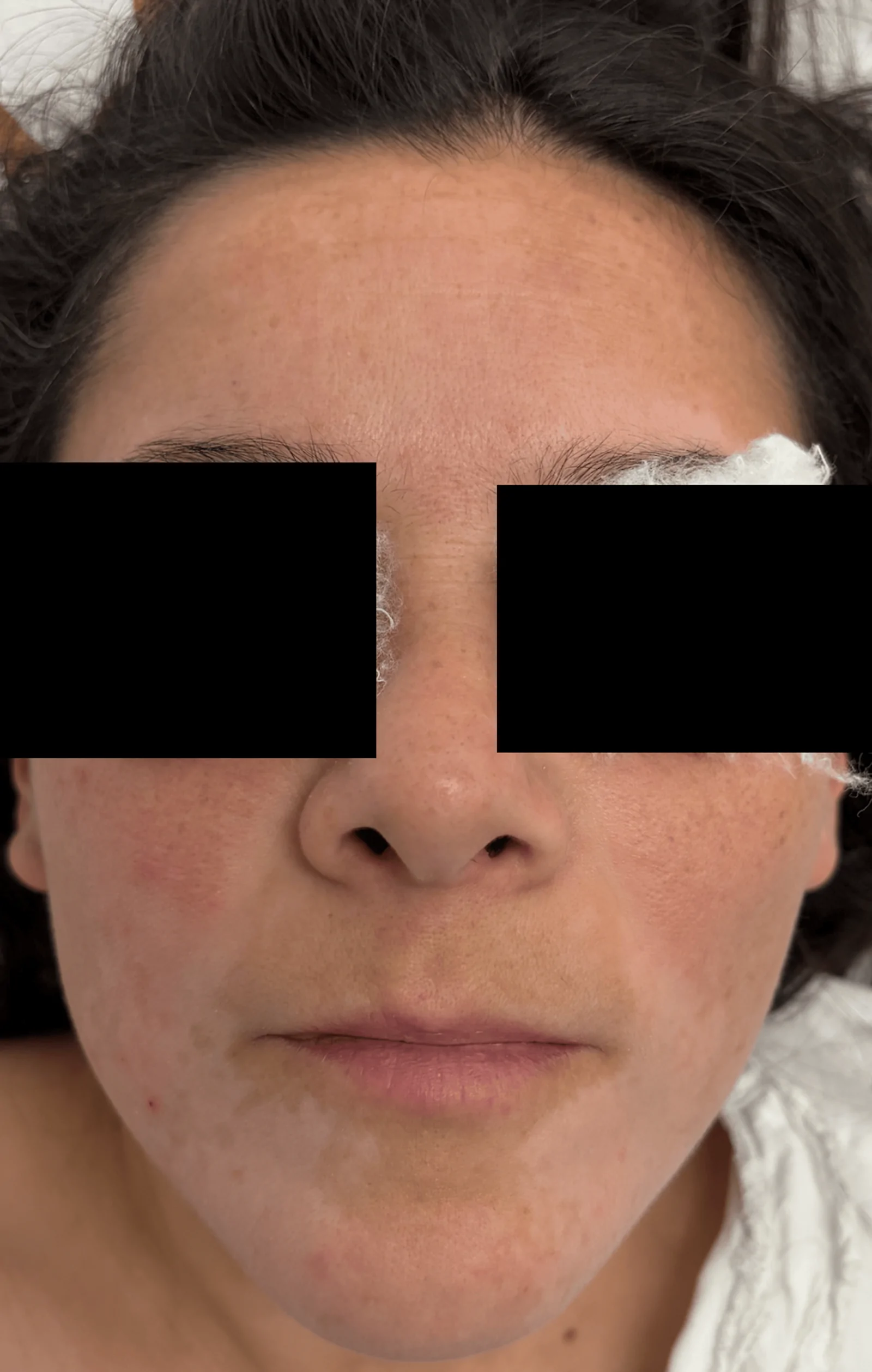
Malar rash Diffuse pink-red erythema and erythematous macules extending from the malar region toward the nasolabial folds with partial sparing of the nasolabial sulcus.

**Table 1:** Dermatologic findings: face

Feature	Description
Color	Faint pink to red erythema, homogeneous tone
Borders	Poorly defined, gradual transition to surrounding normal skin
Surface	Smooth, without evident scaling, crusting, or ulceration
Distribution	Bilateral, symmetric, predominating over malar region and cheeks
Secondary lesions	Absent

Dermatologic Findings: Right Upper Extremity

Physical examination identified a dermatosis on the posterior aspect of the arm (Figure 2), elbow (Figure 3), and shoulder (Figure 4), with a disseminated distribution predominating over periarticular regions and a tendency toward confluence. The elementary lesions consisted of multiple erythematous macules and papules, round to oval in morphology, millimetric in size (1-5 mm), with well-defined borders in the papular lesions and poorly defined, irregular borders in plaques formed by coalescence in the infracondylar periarticular region. The surface was smooth, without scaling, crusting, or ulceration. The predominant color was pink-to-red erythema, most intense over the periarticular region. On palpation, the lesions were soft and non-indurated, without increased local temperature or tenderness. Mild, trace periarticular edema was identified at the elbow (Figure 3), without frank inflammatory signs. No secondary lesions were observed (Table 2).

**Figure 2:**
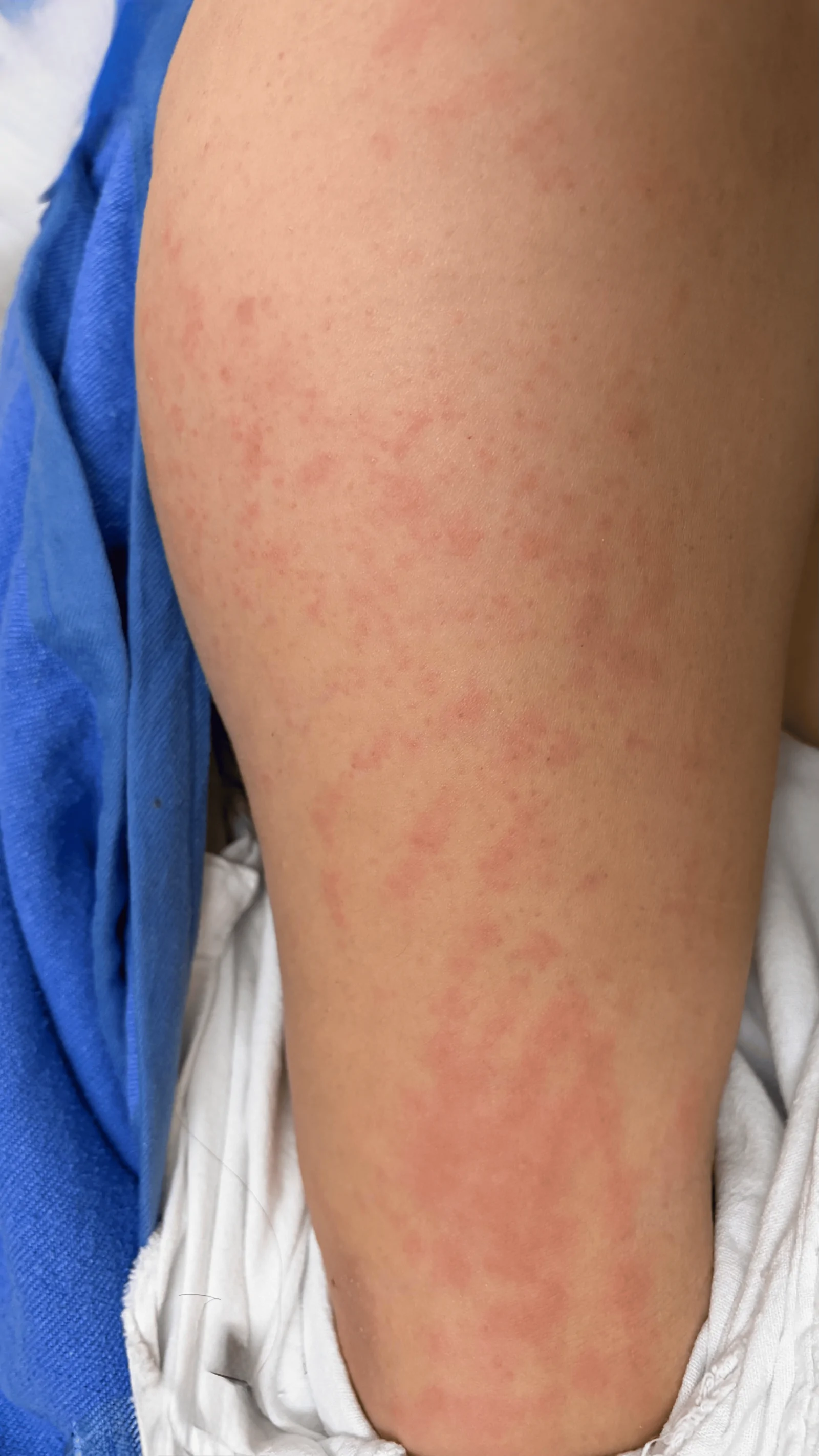
Rash on the right arm Multiple erythematous macules and papules in the lateral aspect of the right arm.

**Figure 3:**
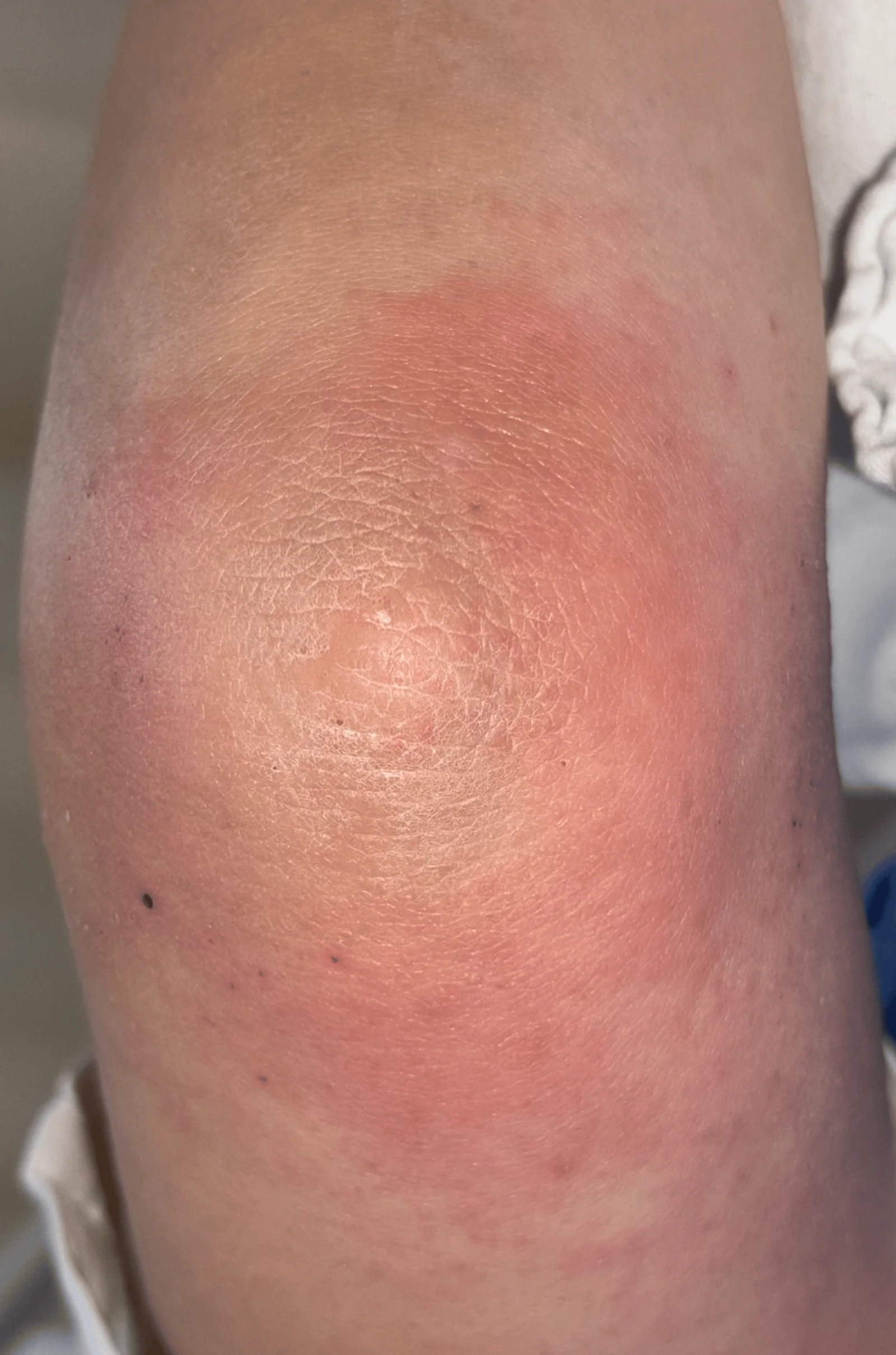
Edema on the right elbow Disseminated, periarticular predominance, erythematous plaque in the posterior aspect of the right elbow.

**Figure 4:**
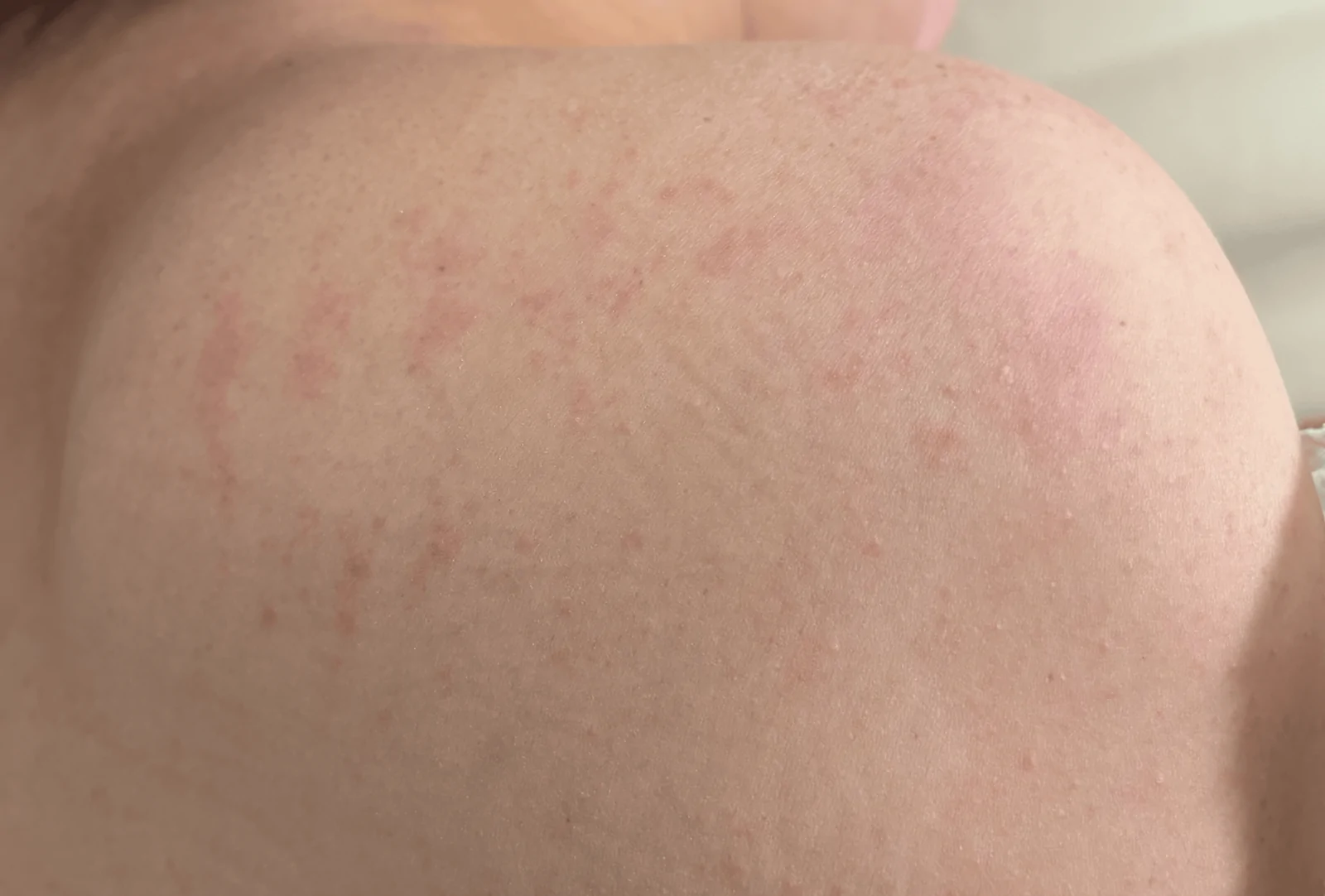
Erythematous macules on the shoulder Multiple on the posterior aspect of the right shoulder, extending to the scapular region.

**Table 2:** Dermatologic findings: right upper extremity

Feature	Description
Location	Posterior aspect of right arm, forearm, elbow, and shoulder
Distribution	Disseminated, periarticular predominance, tendency to confluence
Number	Multiple
Lesion type	Erythematous macules, papules, and plaques
Shape	Round to oval; irregular where coalescent
Size	Millimetric (1–5 mm)
Color	Pink to red erythema
Borders	Well-defined in papules; poorly defined in plaques
Surface	Smooth, without scaling
Consistency	Soft, non-infiltrated, with periarticular edema
Secondary lesions	Absent
Associated findings	Grade +/++++ periarticular edema (infracondylar region)

On later dermatologic re-evaluation, the facial lesions were characterized as areas of low-intensity, slightly perceptible diffuse erythema over normochromic skin, with faint salmon-pink erythematous macules of irregular, confluent morphology, centimetric in extent, poorly defined borders with a gradual and imperceptible transition to surrounding skin, smooth surface without textural change, and no residual lesions, atrophy, postinflammatory pigmentation, or scarring, defining a non-scarring, transient lesion morphology. The cutaneous lesions resolved in parallel with the patient's systemic biochemical improvement, with progressive decline in transaminases, ferritin, and inflammatory markers. No secondary elementary lesions (scale, crust, fissuring, lichenification, atrophy, or scarring) were identified at any point.

Laboratory findings

From the first hospital contact, severe mixed-pattern transaminasemia was documented (R factor 4.8), with a progressive, sustained downward trend over follow-up, an approximately 70% decline in transaminases from peak values by hospital discharge (day 9), though still outside the normal range. Extreme hyperferritinemia was identified, peaking at 19,114.9 ng/mL (approximately 93 times the upper reference limit) before declining by 93% to 1,309.7 ng/mL at the last determination, obtained on outpatient follow-up eight days after discharge, in parallel with clinical improvement. Mild bilineage cytopenia was present on admission, with leukopenia (2.96 × 10³/µL) and mild thrombocytopenia (118 × 10³/µL), with hemoglobin remaining within normal limits (14.6 g/dL; reference range 14.0-18.0 g/dL). The leukopenia was driven predominantly by lymphopenia rather than neutropenia, since the absolute neutrophil count (2.18 × 10³/µL; reference range 2.1-6.1 × 10³/µL) remained within normal limits, with partial recovery of leukocytes and platelets on follow-up, although lymphopenia persisted at the lower limit of the reference range. Inflammatory markers were elevated on admission, with a marked increase in CRP and ESR and only mild elevation of procalcitonin (1.3 ng/mL on day 11), with a trend toward normalization over follow-up, and hypertriglyceridemia persisted throughout follow-up. The thyroid profile showed an isolated elevation of total T4 with normal TSH and free T4, which should be interpreted cautiously given the interactions between severe acute hepatic dysfunction and thyroid hormone metabolism and binding-protein turnover [1]; this isolated total T4 elevation was not pursued further, as it was attributed to hepatic dysfunction-related alterations in thyroid-hormone-binding protein turnover rather than a distinct thyroid disorder, and the patient remained clinically euthyroid throughout admission and at follow-up. The autoimmune panel showed a true-positive antinuclear antibody titer of 1:80 (homogeneous/nuclear pattern) and a borderline anti-smooth-muscle antibody titer of 1:100, with all other lupus-specific and vasculitis-associated antibodies negative. The infectious panel excluded hepatitis A, B, and C, cytomegalovirus, herpes simplex virus, Toxoplasma, and HIV; reactive anti-rubella IgG with negative IgM was consistent with prior immunity rather than acute infection. Table 3 consolidates all laboratory findings.

**Table 3:** Consolidated laboratory findings during hospitalization Hemoglobin ranged between 12.6 and 13.9 g/dL across the early hospitalization period rather than a single admission value. ^†^Thyroid, autoimmune, and infectious panels were single determinations; results are listed under their collection date (day 1, for thyroid and infectious panels, day 6 for the autoimmune panel) rather than serial values. Reference ranges were verified against the institutional laboratory report (Laboratorio Clínico, Hospital General Dr. Darío Fernández Fierro, Institute for Social Security and Services for State Workers (ISSSTE)). ALT: alanine aminotransferase; AST: aspartate aminotransferase; ALP: alkaline phosphatase; LDH: lactate dehydrogenase; GGT: gamma-glutamyl transferase; CRP: C-reactive protein; ESR: erythrocyte sedimentation rate; TSH: thyroid-stimulating hormone; T3: triiodothyronine; T4: thyroxine; Ab: antibody; ANA: antinuclear antibody; anti-dsDNA: anti-double-stranded DNA antibody; anti-Ro/SSA: anti-Ro/Sjögren's-syndrome-related antigen A antibody; anti-RNP: anti-ribonucleoprotein antibody; ANCA-PR3: antineutrophil cytoplasmic antibody-proteinase 3; P-ANCA: perinuclear antineutrophil cytoplasmic antibody; HAV: hepatitis A virus; IgM: immunoglobulin M; HBsAg: hepatitis B surface antigen; CMIA: chemiluminescent microparticle immunoassay; HCV: hepatitis C virus; CMV: cytomegalovirus; IgG: immunoglobulin G; HSV: herpes simplex virus; HIV: human immunodeficiency virus.

Category	Parameter	Reference range	Day 1	Day 2	Day 3	Day 5	Day 6	Day 10	Day 11
Liver panel	ALT (IU/L)	7–52	899	754	808	624	508	264	–
	AST (U/L)	13–39	1063	861	905	548	407	128	–
	ALP (IU/L)	34–104	372	269	268	328	277	–	–
	LDH (U/L)	140–271	2609	2165	2235	1653	1245	–	–
	GGT (U/L)	9–64	453	348	377	482	420	–	–
	Total bilirubin (mg/dL)	0.30–1.00	1.80	–	–	–	–	–	–
	Direct bilirubin (mg/dL)	0.03–0.18	0.73	–	–	–	–	–	–
	R factor	–	4.8	–	–	–	–	–	–
Ferritin	Ferritin (ng/mL)	4.6–204.0	–	–	–	–	19,114.9	–	–
Complete blood count	Leukocytes (×10³/µL)	4.3–10.3	2.96	–	–	–	–	–	–
	Neutrophils (×10³/µL)	2.1–6.1	2.18	–	–	–	–	–	–
	Lymphocytes (×10³/µL)	1.3–3.5	0.65	–	–	–	–	–	–
	Platelets (×10³/µL)	150.0–400.0	118	–	–	–	–	–	–
	Hemoglobin (g/dL)	14.0–18.0	14.6	–	–	–	–	–	–
Inflammatory markers	CRP (mg/dL)	0.0–0.5	–	–	–	1.9	–	–	–
	ESR (mm/h)	0.0–15.0	–	–	–	35	31	–	–
	Procalcitonin (ng/mL)	0.00–0.05	–	–	–	0.51	–	–	1.3
Lipid profile	Triglycerides (mg/dL)	0.0–150.0	–	–	208	–	236	–	–
Coagulation	Fibrinogen (mg/dL)	200–400	–	–	–	–	–	–	349
Thyroid profile^†^	TSH (µIU/L)	0.4–4.9	3.8	–	–	–	–	–	–
	Total T3 (ng/mL)	0.64–1.52	1.34	–	–	–	–	–	–
	Free T3 (pg/mL)	1.58–3.91	2.73	–	–	–	–	–	–
	Total T4 (µg/dL)	4.87–11.72	14.52	–	–	–	–	–	–
	Free T4 (ng/dL)	0.700–1.480	1.23	–	–	–	–	–	–
	TSH-receptor Ab (IU/L)	Negative ≤1.75	1.12	–	–	–	–	–	–
Autoimmune panel^†^	ANA, titer/pattern	Negative <1:40	–	–	–	–	–	–	–
	Anti-smooth-muscle Ab	Negative <1:100	–	–	–	–	–	–	–
	Anti-dsDNA (IU/mL)	Negative <100	–	–	–	–	–	–	–
	Anti-Ro/SSA	Negative <20	–	–	–	–	–	–	–
	Anti-Smith (CU)	Negative <20	–	–	–	–	–	–	–
	Anti-RNP	1.1–4.4	–	–	–	–	–	–	–
	ANCA-PR3 (UR/mL)	Negative <20	–	–	–	–	–	–	–
	P-ANCA (UR/mL)	Negative <20	–	–	–	–	–	–	–
Infectious panel^†^	Anti-HAV IgM	Non-reactive 0.0–0.80	0.150 (non-reactive)	–	–	–	–	–	–
	HBsAg (CMIA)	Non-reactive 0.0–1.0	0.35 (non-reactive)	–	–	–	–	–	–
	Anti-HCV (CMIA)	Non-reactive 0.0–1.0	0.090 (non-reactive)	–	–	–	–	–	–
	CMV IgG / IgM	IgG <6.0; IgM 0.0–0.84 non-reactive	IgG 0.50, IgM 0.23 (both non-reactive)	–	–	–	–	–	–
	Toxoplasma IgG / IgM	IgG <1.6; IgM <0.50 non-reactive	IgG 0.10, IgM 0.07 (both non-reactive)	–	–	–	–	–	–
	HSV IgG / IgM	IgG negative <0.9; IgM negative 0–0.89	IgG 0.10, IgM 0.50 (both negative)	–	–	–	–	–	–
	HIV	Non-reactive	Non-reactive	–	–	–	–	–	–
	Rubella IgG / IgM	IgG non-reactive 0.0–4.9 (reactive >10); IgM non-reactive <1.20	IgG 41.70 (reactive = immunity), IgM 0.20 (non-reactive)	–	–	–	–	–	–

Imaging

Abdominal ultrasound. The spleen was normal in shape and position, measuring 122 × 61 mm in its longitudinal and anteroposterior axes, with homogeneous parenchyma. The hepatorenal and perisplenic spaces showed no abnormalities, and no cholelithiasis was identified. Findings were suggestive of grade I hepatic steatosis, with splenomegaly of undetermined origin requiring clinical-radiologic correlation.

Skin biopsy

A skin biopsy of the posterior aspect of the right elbow was performed, which demonstrated spongiotic dermatosis with interface vacuolar degeneration and a subcorneal blister pattern (Figure 5).

**Figure 5:**
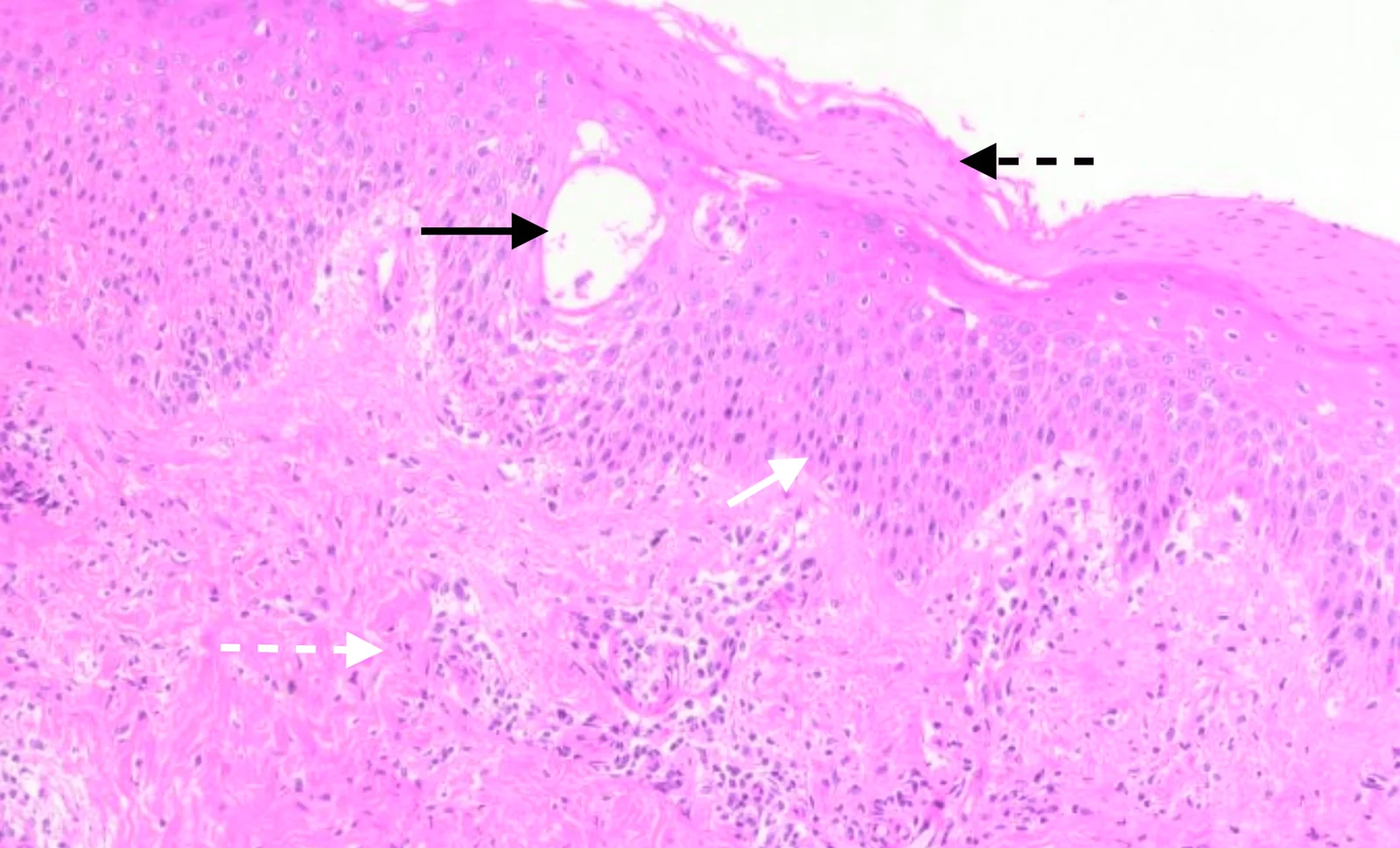
Spongiotic dermatosis with interface vacuolar degeneration and a subcorneal blister pattern Epidermis with hyperparakeratosis (black dotted arrow) and subcorneal blistering (black arrow), pseudoepitheliomatous hyperplasia with acanthosis and spongiosis underlying the blisters with interface vacuolar degeneration (white arrow) and a perivascular lymphocytic infiltrate in the reticular dermis without vasculitis (white dotted arrow).

The diagnostic process was complicated by numerous factors: a nonspecific initial presentation with fever, abdominal pain, and cytopenias; a mixed hepatic enzyme pattern that made it difficult to establish a toxic, infectious, or autoimmune etiology; negative viral serologies; a true-positive but low-titer antinuclear antibody; extreme hyperferritinemia (19,114.9 ng/mL); and exposure to a supplement (Ismerely FTX Plus) subsequently confirmed via a public FDA notice to be adulterated with dexamethasone phosphate and diclofenac, ingredients not declared on the product label.

## Discussion

Non-dermatologist physicians often have limited training in characterizing cutaneous lesions and interpreting histopathological findings from skin biopsies. Although skin biopsy is highly useful for diagnostic orientation, it must be interpreted within the context of clinical manifestations, as histopathological findings are rarely exclusive to a single nosologic entity. Thorough evaluation of cutaneous manifestations is valuable for the differential diagnosis of systemic conditions, particularly in severe acute hepatic involvement, to avoid overlooking immunologic entities that may affect long-term health.

Dermatologic manifestations and histopathologic findings

A skin biopsy was performed as part of the differential diagnostic approach between atopic dermatitis and cutaneous lupus erythematosus. The histopathologic study reported a spongiotic dermatosis with interface vacuolar degeneration and a subcorneal blister pattern, with a predominantly lymphoid inflammatory infiltrate and no vasculitis.

Morphologically, interface vacuolar degeneration is a classic finding of immune-mediated injury at the dermoepidermal junction, commonly seen in cutaneous lupus erythematosus, dermatomyositis, and interface-pattern (lichenoid) drug reactions, which can range from mild eruptions to severe, progressive forms if the causative agent is not withdrawn [7]. However, the coexistence of spongiosis and subcorneal blistering brings an eczematous component that widens the differential toward reactive dermatoses and drug eruptions.

A particularly relevant feature was the absence of eosinophils in the inflammatory infiltrate, which carries substantial discriminative value. In DRESS (Drug Reaction with Eosinophilia and Systemic Symptoms), tissue and peripheral eosinophilia are characteristic findings, reflecting drug-induced Th2 activation [5,6]. In this case, both the histology and the complete blood count (without significant eosinophilia) made this diagnosis unlikely, as also noted explicitly in the pathology report.

Clinicopathologic correlation

Integrating the clinical and histopathologic dermatologic findings with the patient's systemic context, including bilateral malar erythema, a true-positive antinuclear antibody (ANA) at a 1:80 titer with a homogeneous/nuclear pattern, borderline-positive anti-smooth muscle antibodies (1:100), interface vacuolar degeneration on biopsy, severe transaminasemia, extreme hyperferritinemia, cytopenias, and splenomegaly, could, at first glance, suggest subacute cutaneous lupus erythematosus with possible systemic involvement. However, several clinical and serologic elements weaken this possibility: the patient had negative disease-specific autoantibodies (anti-double-stranded DNA (anti-dsDNA), anti-Ro/Sjögren syndrome-related antigen A (anti-Ro/SSA), anti-Smith, anti-ribonucleoprotein (anti-RNP), antineutrophil cytoplasmic antibodies (ANCA)) and lacked the systemic manifestations typically required to fulfill lupus classification criteria. A low-positive ANA titer, as in this patient, has limited positive predictive value for an underlying systemic autoimmune disease and must be weighed with titer, pattern, and clinical context rather than interpreted as a standalone diagnostic finding [9]. This clinicopathologic discordance is well recognized in dermatopathology, where interface patterns are not exclusive to autoimmune disease and can also be observed in idiosyncratic drug reactions.

In this context, the most coherent interpretation is that the cutaneous lesions form part of a spectrum of drug- or supplement-induced interface dermatitis. This reaction shares immunologic mechanisms with idiosyncratic hepatotoxicity, particularly activation of cytotoxic T lymphocytes directed against keratinocytes and hepatocytes modified by drug metabolites, in this case, the undisclosed dexamethasone and diclofenac identified in the ingested supplement. This hypothesis unifies the cutaneous and hepatic findings within a single systemic immunologic process triggered by the adulterated product. Additionally, the subcorneal blistering suggests more advanced epidermal injury, likely related to proinflammatory cytokine release and disruption of intercellular cohesion, a nonspecific but recognized feature of severe drug eruptions and acute cutaneous inflammation.

Differential diagnosis: atopic dermatitis, lupus erythematosus, and drug eruptions

There are many characteristics of the lesions that must be evaluated in order to differentiate atopic dermatitis, systemic lupus erythematosus, cutaneous (subacute/discoid) lupus erythematosus, and drug eruptions, principally clinical, distributional, histologic, immunologic, and prognostic features (Table 4).

**Table 4:** Comparative features of atopic dermatitis, systemic lupus erythematosus, cutaneous lupus erythematosus, and drug eruptions SLE: systemic lupus erythematosus; SCLE: subacute cutaneous lupus erythematosus; DRESS: drug reaction with eosinophilia and systemic symptoms; SJS: Stevens-Johnson syndrome; TEN: toxic epidermal necrolysis; DIF: direct immunofluorescence; IgE: immunoglobulin E; RAST: radioallergosorbent test; ANA: antinuclear antibody; anti-dsDNA: anti-double-stranded DNA antibody; anti-Sm: anti-Smith antibody; C3: complement component 3; C4: complement component 4; anti-Ro/SSA: anti-Ro/Sjögren's-syndrome-related antigen A antibody; EASI: Eczema Area and Severity Index; SCORAD: Scoring Atopic Dermatitis; IGA: Investigator's Global Assessment; SLICC: Systemic Lupus International Collaborating Clinics; ACR: American College of Rheumatology; EULAR: European Alliance of Associations for Rheumatology; SLEDAI: Systemic Lupus Erythematosus Disease Activity Index; CLASI: Cutaneous Lupus Erythematosus Disease Area and Severity Index; ALDEN: Algorithm of Drug Causality for Epidermal Necrolysis; IL-4/13: interleukin 4/interleukin 13; NSAIDs: nonsteroidal anti-inflammatory drugs; IVIG: intravenous immunoglobulin.

Category	Atopic Dermatitis	Systemic Lupus Erythematosus	Cutaneous Lupus Erythematosus	Drug Eruptions
Primary lesions	Erythematous papules, vesicles, crusts; chronic lichenified eczema	Diffuse malar erythema; oral ulcers; photosensitive rash	Erythematous, scaly plaques with atrophy and hyperpigmentation; telangiectasias	Variable: maculopapular, bullous, or pustular exanthem depending on type
Distribution	Flexural areas (popliteal, antecubital fossae), face, neck	Face, upper trunk, hands (photodistributed); malar region	Face, upper trunk, and photoexposed areas (V-neckline)	Generalized (maculopapular); localized to contact site (fixed drug eruption)
Symptoms	Chronic, often intense pruritus; excoriations	Mild pain/burning; fixed malar erythema sparing nasolabial folds	Variable pruritus; thick scale; burning sensation in active lesions	Pruritus, burning, urticaria; severe pain in bullous reactions
Systemic involvement	Asthma, rhinitis (atopic triad); no direct organ involvement	Nephritis, serositis, arthritis, neuropathy, cytopenias, antiphospholipid syndrome	Generally limited to skin; ~5% progression to SLE (SCLE)	DRESS: fever, lymphadenopathy, hepatitis, eosinophilia; SJS/TEN: mucosal involvement, sepsis risk
Histology	Spongiosis, perivascular lymphocytic infiltrate, hyperkeratosis, acanthosis	Interface dermatitis, mucin deposition, basement membrane thickening (DIF+)	Interface dermatitis, follicular plugging, epidermal atrophy, lichenoid infiltrate	Spongiosis (eczematous pattern); keratinocyte necrosis (SJS/TEN); eosinophils (DRESS)
Immunology/labs	Elevated total IgE; eosinophilia; positive RAST/prick testing; filaggrin mutation in severe forms	ANA+, anti-dsDNA+, anti-Sm+; low C3/C4; proteinuria; cytopenias	ANA+ (low titer); anti-Ro/SSA+ (SCLE); antihistone antibodies (drug-induced lupus)	Eosinophilia (DRESS); positive patch/provocation test; lymphocyte transformation test; drug-specific IgE
Diagnostic criteria	Hanifin and Rajka; EASI; SCORAD; IGA	SLICC 2012/ACR-EULAR 2019 (≥10 points); SLEDAI for activity	Gilliam classification (acute/subacute/chronic); biopsy + DIF; CLASI	Drug-eruption chronology; Naranjo score; RegiSCAR/ALDEN score (DRESS/TEN); biopsy
First-line treatment	Emollients; topical corticosteroids; calcineurin inhibitors; dupilumab (anti-IL-4/13)	Hydroxychloroquine; NSAIDs; corticosteroids; mycophenolate mofetil, azathioprine, belimumab	Photoprotection; antimalarials; topical/intralesional corticosteroids; dapsone (SCLE)	Discontinue causative drug; corticosteroids (DRESS); cyclosporine or IVIG (SJS/TEN)
Course/prognosis	Chronic, relapsing; improvement in adolescence in ~60%; quality of life affected	Chronic with flares; cumulative organ damage; increased cardiovascular morbidity/mortality	Good in limited forms; risk of scarring and alopecia in discoid variant	Self-limited upon drug withdrawal; SJS mortality ~5%, TEN up to 35%

Extreme hyperferritinemia and probable secondary hemophagocytic syndrome

The marked initial hyperferritinemia (19,114.9 ng/mL), together with fever, cytopenias, splenomegaly, hypertriglyceridemia, and elevated lactate dehydrogenase (LDH), is consistent with macrophage activation compatible with secondary hemophagocytic lymphohistiocytosis. A calculated HScore using the data available in this case, no known immunosuppression (0 points), maximum temperature of 39.4°C (33 points), splenomegaly without hepatomegaly (23 points), one qualifying cytopenia lineage by strict HScore cutoffs (0 points), ferritin >6,000 ng/mL (50 points), triglycerides 236 mg/dL/≈2.67 mmol/L (44 points), fibrinogen 349 mg/dL/≈3.49 g/L, above the 2.5 g/L cutoff (0 points), aspartate aminotransferase (AST) ≥30 IU/L (19 points), and no bone marrow aspirate performed to assess for hemophagocytosis (0 points), yielded a total of 169, corresponding to an estimated 40% probability of hemophagocytic lymphohistiocytosis (HLH).

Applying the HLH-2004 diagnostic criteria, this patient met 3 of the 8 items: fever ≥38.5°C (39.4°C), splenomegaly, and ferritin ≥500 µg/L (19,114.9 ng/mL), while none of the individual cytopenia thresholds were met (hemoglobin 14.6 g/dL, absolute neutrophil count 2.18 × 10³/µL, and platelet count 118 × 10³/µL all remained above the HLH-2004 cutoffs), and neither hypertriglyceridemia ≥265 mg/dL nor hypofibrinogenemia ≤150 mg/dL was present (triglycerides 236 mg/dL; fibrinogen 349 mg/dL). The remaining three criteria (bone marrow, spleen, or lymph node hemophagocytosis; NK-cell activity; soluble CD25) were not assessed. Because only 3 of the 8 HLH-2004 criteria were fulfilled, well short of the 5 required for a formal diagnosis, and several relevant tests were unavailable, this case is best characterized as probable rather than confirmed secondary HLH.

Current consensus guidance for adults emphasizes that extreme hyperferritinemia in this range, combined with cytopenias and organomegaly, is designed to prompt active screening for HLH even in the absence of a full diagnostic score or complete criterion panel. Identifying and removing the underlying trigger, infectious, autoimmune, malignant, or drug-related, is central to management [3].

Given that infection is the leading trigger to consider in the differential diagnosis of secondary HLH, an infectious contribution was carefully assessed in this patient. Procalcitonin was only mildly elevated (1.3 ng/mL), a level more consistent with a sterile systemic hyperinflammatory process than with bacterial sepsis, in which procalcitonin typically rises to substantially higher levels. Blood and urine cultures were not obtained, as the patient had no localizing signs of infection, no hemodynamic instability, and no clinical deterioration suggestive of a septic process at any point during admission; furthermore, the extended infectious serology panel (hepatitis A, B, and C, cytomegalovirus, herpes simplex virus, Toxoplasma, and HIV) was negative, and the clinical course improved in parallel with withdrawal of the causative supplement alone, without antimicrobial therapy. Taken together, these findings argue against an infectious trigger and support the adulterated supplement as the primary driver of the hyperinflammatory state in this case.

In this patient, the most probable trigger was the combination of severe hepatocellular injury, with release of stored hepatic ferritin, and an exaggerated systemic inflammatory response to the adulterated supplement, occurring in a host with pre-existing immune dysregulation related to hypothyroidism and atopic dermatitis. The spontaneous, progressive resolution of hyperferritinemia, cytopenias, and hypertriglyceridemia after withdrawal of the causative agent, without immunosuppressive therapy, argues against a primary autoimmune or malignancy-driven process and supports a reactive, drug-triggered mechanism [4].

## Conclusions

This case illustrates how clinicopathologic correlation, rather than reliance on any single serologic, biochemical, or histopathologic finding, was essential to resolve a complex diagnostic challenge involving overlapping features of atopic dermatitis, cutaneous lupus erythematosus, and drug-induced interface dermatitis in the setting of severe hepatotoxicity and probable secondary hemophagocytic syndrome. Borderline autoantibody positivity and biopsy findings suggestive of an interface dermatitis pattern were not, by themselves, sufficient to establish a diagnosis of cutaneous or systemic lupus erythematosus, particularly given the absence of disease-specific autoantibodies, the absence of tissue eosinophilia arguing against DRESS, and the spontaneous resolution of all findings after withdrawal of the causative agent alone.

Histopathological findings on skin biopsy are valuable in the differential diagnosis of immune-mediated diseases with suspected systemic involvement, provided they are interpreted within the appropriate clinical context. Further studies are needed to improve the clinical and histopathological differentiation between subacute cutaneous lupus erythematosus and other immune-mediated dermatitides, as these entities may share clinical and histopathological manifestations while carrying different prognoses and treatments.

This case further highlights adulterated over-the-counter dietary and joint supplements as an underrecognized cause of combined hepatic and cutaneous immune-mediated injury. It accentuates the relevance of considering hidden pharmaceutical adulterants, in this case, undisclosed dexamethasone and diclofenac, when evaluating patients with unexplained severe hepatotoxicity, hyperferritinemia, and cutaneous eruptions following supplement use. Close multidisciplinary follow-up with dermatology, rheumatology, and hepatology is fundamental in such cases to confirm disease resolution and to detect any evolution toward a defined autoimmune condition over time.
